# The ABCC6 Transporter: A New Player in Biomineralization

**DOI:** 10.3390/ijms18091941

**Published:** 2017-09-11

**Authors:** Guillaume Favre, Audrey Laurain, Tamas Aranyi, Flora Szeri, Krisztina Fulop, Olivier Le Saux, Christophe Duranton, Gilles Kauffenstein, Ludovic Martin, Georges Lefthériotis

**Affiliations:** 1 FINSERM, U 1081, Aging and Diabetes Team, Institute for Research on Cancer and Aging of Nice (IRCAN), 06107 Nice, France; 2 CNRS, UMR7284, Institute for Research on Cancer and Aging of Nice (IRCAN), 06107 Nice, France; 3 Faculty of Medicine, University of Nice-Sophia Antipolis, 06107 Nice, France; leftheriotis.g@chu-nice.fr; 4 Nephrology Department, University Hospital, 06107 Nice, France; a.laurain@live.fr; 5 Institute of Enzymology, Research Centre for Natural Sciences, Hungarian Academy of Sciences, 1117 Budapest, Hungary; aranyi.tamas@ttk.mta.hu (T.A.); floraszeri@me.com (F.S.); fulop.krisztina@ttk.mta.hu (K.F.); 6 Department Cell and Molecular Biology, John A. Burns School of Medicine, University of Hawaii, Honolulu, HI 96813, USA; lesaux@hawaii.edu; 7 Laboratory of Physiology and Molecular Medicine (LP2M) UMR CNRS 7073, 06107 Nice, France; duranton@unice.fr; 8 UMR CNRS 6015-Inserm 1083, School of Medicine, Bretagne Loire University, 49045 Angers, France; gilles.kauffenstein@gmail.com (G.K.); LuMartin@chu-angers.fr (L.M.); 9 PXE Health and Research Center, University Hospital of Angers, 49045 Angers, France

**Keywords:** ABC transporter, inorganic pyrophosphate, pseudoxanthoma elasticum, arterial calcifications, chronic kidney disease

## Abstract

Pseudoxanthoma elasticum (PXE) is an inherited metabolic disease with autosomal recessive inheritance caused by mutations in the *ABCC6* gene. Since the first description of the disease in 1896, alleging a disease involving the elastic fibers, the concept evolved with the further discoveries of the pivotal role of ectopic mineralization that is preponderant in the elastin-rich tissues of the skin, eyes and blood vessel walls. After discovery of the causative gene of the disease in 2000, the function of the ABCC6 protein remains elusive. More than 300 mutations have been now reported and the concept of a dermal disease has progressively evolved toward a metabolic disorder resulting from the remote effects caused by lack of a circulating anti-mineralization factor. Very recently, evidence has accumulated that this anti-mineralizing factor is inorganic pyrophosphate (PPi). This leads to decreased PPi/Pi (inorganic phosphate) ratio that results from the lack of extracellular ATP release by hepatocytes and probably renal cells harboring the mutant ABCC6 protein. However, the mechanism by which ABCC6 dysfunction causes diminished ATP release remains an enigma. Studies of other ABC transporters, such as ABCC7 or ABCC1 could help our understanding of what ABCC6 exact function is. Data and a hypothesis on the possible roles of ABCC6 in acquired metabolic diseases are also discussed.

## 1. Introduction

Membrane transporters are basic cellular elements, which play a central role in various cellular functions and cells’ integrity. Adenosine triphosphate (ATP)-binding cassette (ABC) transporters are a large family of 48 members involved in the pathogenesis of various inherited metabolic diseases. In this regard, ABCC6 is one of these new players, whose deficiency is responsible for pseudoxanthoma elasticum (PXE), a rare and intriguing inherited disease with unexplained female 2 third prevalence and whose phenotype is characterized by ectopic calcifications in elastin-rich tissues such as the skin, the Burch’s membrane of the retina and the arterial wall. To date, the physiological role of ABCC6 and its preferential substrate(s) remain an enigma. Recent findings are now showing a pivotal role in the regulation of extracellular nucleotid metabolism and in the direct and remote control of biomineralization. This review will summarize the current knowledge and speculated roles of ABCC6 transporter not only in PXE, but also in frequent acquired metabolic diseases characterized by arterial calcifications (AC).

## 2. Pseudoxanthoma Elasticum: The Story of a “misnamed” Disease

The study of PXE, and thus the discovery of ABCC6, is paved by important clinical and scientific rebounds. Thus a brief time-sketch of the progresses in our knowledge and on-going debates are mandatory to the understanding of its biological and clinical relevance. PXE (also known as Grönblad-Strandberg syndrome, elastosis dystrophica or elastodysplasia calcificans) is a rare inherited disease (OMIM 264800, prevalence 1/25,000 to 1/50,000) characterized by a generalized accumulation of calcified and fragmented elastic fibers selectively affecting the elastin-rich tissues such as the skin, the retina and the vascular wall. Organs such as the kidneys, joints and tendons are affected to various degrees, but remarkably, the pulmonary tissue seems to be spared in the disease phenotype [1,2]. The skin manifestations are often the initial and most visible lesions in PXE, with a “pseudoxanthoma” aspect affecting, for yet unknown reasons, the flexural areas of the body (i.e. neck, arm, knee joints, groin) and periumbilical areas. The ambiguous name “pseudoxanthoma” arose from the fact that the skin lesions mimic “xanthoma” lesions (i.e. subcutaneous papules of yellow-orange color). Following the initial and independent reports on “xanthomas” lesion [3,4,5], François-Ferdinand Darier in 1896 presented the idea that they were distinct from xanthomas with an unusual involvement of elastic tissues, thereafter introducing the prefix “pseudo” as to cast doubt on the real nature of the dermal lesions [6]. The fundamental histopathological feature of PXE, i.e. the calcification of elastin, were described first in 1901 by von Tannenhain; Werther seemed to have seeded the idea that PXE was a congenital trait [7]. Knapp was the first to report a specific retinopathy coining the descriptor “angioid streaks”, still a pathognomonic clinical manifestation of the disease. Finally, in 1929, Grönblad, an opthalmologist and Strandberg, a dermatologist, determined that PXE was a specific entity. Currently, PXE diagnosis relies on the following criteria: clinically suggestive skin lesions, angioid streaks, fragmented and calcified elastic fibers on skin biopsy [8]. The cardiovascular complications linked to PXE were initially reported by Balzer in 1884 who described an "elastic degeneration of the skin and heart" and Carlsborg gave the first well-documented study of cardiovascular elastic tissue calcification in 1944. Since that time, the term “pseudoxanthoma elasticum” continues to be the most frequently used to designate this pathology in the literature for historical reasons. However, one would suggest elasto-calcinosis as more appropriate [9].

## 3. Vascular Lesions of PXE

Beside the skin and retinal lesions, the arterial phenotype is an important aspect of the disease. The internal elastic laminae in arteries are the most frequently affected. The small and medium peripheral arterial beds are mostly involved [10], although other arteries, such as the coronary and the cerebral arteries may also be affected. Typically, arterial lesions are characterized by an obstruction of the lumen from a diffuse thickening of the wall and an echogenic plaque revealing calcification. The lesions are primarily found on straight segments in leg arteries such as the superficialis femoralis or arteries below knee. To date, the exact nature of these lesions is unknown in humans and lower limb intermittent claudication due to peripheral occlusive disease represent a typical symptom of the disease affecting more than 20% of the patients [11]. Contrary to what is expected with arterial mediacalcosis, arterial compressibility is well-preserved in PXE, suggesting a specific arterial remodeling [11]. In fact, abnormal proteoglycan deposits, authenticated by post mortem histology, contributing to the arterial obstruction was also advocated to preserve the compressibility of the peripheral arteries despite fragmentation and calcifications of the elastin fibers [12,13]. To the best of our knowledge, medial hyperplasia has never been reported in these patients. Arterial wall aneurysms have been described in PXE patients, mostly in the intracranial vasculature and as an indirect consequence of the collateral flow [14]. Although PXE patients are at high risk for stroke, it is mostly attributed to a small-cerebral vessel disease with narrowed arteries rather than to embolisms. Increased myogenic tone in resistance arteries, primarily evidenced in *Abcc6*^−/−^ mice [15], may be responsible for such ischemic events, even no evidence for such mechanisms have been shown in humans. Taken as a whole, the vascular phenotype of PXE is unique and is not associated to bone mineralization defect [16] in contrast with non-genetic vascular calcifying diseases, namely osteoporosis and/or chronic kidney disease (CKD) [17].

Although arterial calcifications represent the most visible cardiovascular lesions in PXE, other abnormalities have been reported [9]. Cardiac tissue may be affected in PXE. Some cohort studies have reported specific changes in myocardial function whereas others reported that preclinical cardiac dysfunction were rare [18,19]. Recently, results from prospective and comprehensive assessment of cardiac function in 67 PXE patients aged 48 ± 15 years were found not to be different from those of a comparable population regarding coronary artery disease, valve disease and echocardiography findings [20].

## 4. Lessons Learned from Knockout Mice

Knockout animal models mimicking PXE disease have been developed using targeted disruption of selected exons in *Abcc6* [21,22]. These models replicate the human phenotype with mouse-specific distinctions. For example, *Abcc6*^−/−^ mice lack skin lesions but exhibit abnormal calcification of the capsule of the vibrissae, a connective tissue absent in humans [21]. These knockout mice also mimic the human cardiovascular phenotype [21,23] including higher intima-media thickness [24]. A higher myogenic tone in distal arteries has been also observed [15], which has not been reported yet in PXE patients. Finally, cardiac hypertrophy was described in *Abcc6*^−/−^ mice after 24 months of life, suggesting that late cardiomyopathy could develop in older PXE patients [20]. *Abcc6* deficiency was recently linked to an acute cardiac calcification phenotype referred to as dystrophic cardiac calcification (DCC) in several inbred strains of mice including C3H/HeJ and DBA/2J [25,26,27,28,29]. DCC is an autosomal recessive trait that was described several decades ago [27,29] in mice but not in humans to our knowledge. It is a spontaneous condition affecting cardiovascular tissues that can also be triggered into an acute phenotype by specific diet or direct injury causing myocardia necrotic lesions [27,29]. The *Dyscalc1* locus affecting DCC was mapped to chromosome 7 [30,31,32] and a single *Abcc6* gene mutation leads to a constitutive decrease in protein levels in the liver [25,28]. Additional loci affecting the penetrance and expression of the DCC phenotype were mapped to chromosomes 4, 12, and 14 [31]. Other knock out models have been engineered in rats [33] and zebrafish [34] showing the importance of the *Abcc6* gene in the calcifying phenotype of vertebrae.

## 5. Molecular Aspects of ABCC6

**Figure 1:**
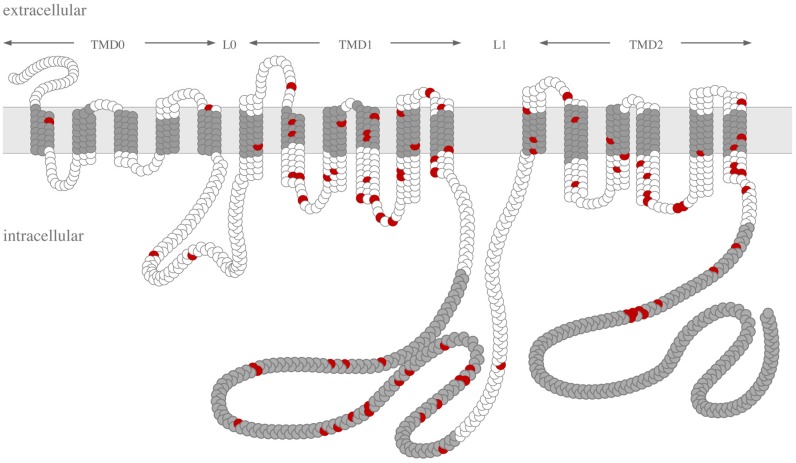
Topology model illustrating the distribution of PXE-associated missense mutations in the ABCC6 protein. Legend: nucleotidE binding domains and the transmembrane helices are shown in grey. Missense mutations are represented in red. The transmembrane domains (TMD0, TMD1, and TMD2) and the linker regions (L0, L1) of the protein are indicated.

In 2000, Le Saux et al. identified the first PXE-causing mutations in the gene encoding ABCC6 (also referred to as multi-drug resistance-associated protein-6, MRP6) [35]. Genetic linkage analysis failed to reveal any locus heterogeneity, suggesting that *ABCC6* is solely responsible for PXE. Today, more than 300 mutations have been identified (Table 1 and Figure 1). The vast majority is represented by single nucleotide substitutions resulting in missense, nonsense and splice variants while a few others are large and small deletions or insertions [36]. The *ABCC6* gene is a member of the gene family encoding ATP-binding cassette (ABC) proteins, one of the largest gene families. The 48 human ABC transporters are distributed in 7 classes from A to G. All these genes code for proteins, which have conserved domains specific to ATP binding and hydrolysis (e.g., Walker A, B and signature sequence). All ABC transporters are transmembrane proteins as their function mainly relate to the cellular efflux of a variety of compounds. ABC transporters are composed of transmembrane domain (TMD) and nucleotide binding domain (NBD). Two NDB domains interact to utilize ATP. The ABCC transporters are divided into two subgroups based on their short or long structure. The long ABCC family members, ABCC1, 2, 3, 6 and 10 contain a specific N-terminal TMD0 domain followed by an L0 linker segment connecting to the transmembrane and NDB-1 and -2 regions. Genes coding for long ABCC proteins are closely related to each other and are present in all eukaryotic kingdoms. They are likely to have evolved from a distant ancestor by a series of gene duplication events [37]. The evolution of ABC transporters is still ongoing. Indeed, the human *ABCC6* underwent several recent partial gene duplications leading to two pseudogenes and a third shorter fragment [38]. These pseudogenes appeared by similar mechanisms in different primates but independently in humans and chimpanzee [37].

The transcriptional regulation of *ABCC6* in human and mice has been investigated in detail. Both activator and silencer elements have been identified in the promoter [39]. The core promoter was mapped to the −145/+72 bp (numbering relative to the translational start site), which confers a ubiquitous low expression in cell assay systems [39]. Two other activator sequences were identified in the upstream promoter region: an evolutionarily highly conserved region between −209/−145 bp and a stronger primate-specific region between −234 and −209 bp. Both of these elements confer tissue-specific activity to the gene [40,41]. Furthermore, a strong primate-specific activator sequence was identified in the first intron of the gene. This intronic sequence interacts with and its activity depends on regulatory sequences of the promoter [42]. Several transcription factors playing an important role in the regulation of *ABCC6* expression were identified. The most important among them is hepatocyte nuclear factor 4α (HNF4α), which binds to the evolutionarily highly conserved element of the promoter [40,41,42]. HNF4α plays a particularly important role in the maintenance of homeostasis of hepatocytes by regulating the expression of more than 1000 genes. This nuclear receptor seems to be a major determinant of tissue-specificity for *ABCC6* as this gene is highly expressed in tissues and cells in which HNF4α is present. In another set of experiments the sensitivity of *ABCC6* expression to environmental stimuli has been demonstrated and the molecular mechanisms of the alteration of gene expression level was also elucidated in some cases. The expression level of the gene is down regulated by growth factors (hepatocyte growth factor, epidermal growth factor and potentially transforming growth factor-β, although the latter is controversial) [40,43] and oxidative stress. Several studies have shown the inhibitory effect of oxidative stress on *ABCC6* expression [12,40], which led to the hypothesis that, this could contribute to development of PXE-like manifestations in beta-thalassemia patients [44,45]. It has been shown that these effects, in part converge on the ERK1/2 pathway, which upon activation phosphorylates HNF4α leading to the reduced binding of the factor to its response element in the promoter [46]. This may then cause the repression of *ABCC6* expression. Similarly, other environmental stimuli activating protein kinase cascades (PKA, PKC and AMPK) lead to the reduced activity of HNF4α and the decreased expression of *ABCC6*. ABCC6 was reported to be capable to transport leukotriene C_4_ (LTC_4_) and *N*-ethylmaleimide *S*-glutathione (NEM-GS) in vitro [47].

## 6. Pathophysiology of ABCC6

The expression pattern of *ABCC6* gene has been extensively studied. *ABCC6* is primarily expressed in the liver [51,52,53,54,55], with moderate levels in the proximal tubules of the kidneys [56,57] and has also been detected in the intestines [42,53]. While the expression level is considered to be low or absent in other tissues, few studies reported ubiquitous presence of ABCC6 [52,58]. The paradox that ABCC6 is almost entirely missing from tissues directly affected by ectopic mineralization in humans, mice and rats [33,59,60] has led to the hypothesis that PXE could be due to a circulating factor [61]. Accordingly, a systemic factor transported by ABCC6 into the circulation under physiological conditions, would play a key role in preventing ectopic calcification. This hypothesis was supported by parabiotic experiments using crosses of wild type and *Abcc6^−/−^* mice with *Rag*^−/−^ immune-tolerant animals. Indeed, this study found that the calcifying phenotype of *Abcc6^−/−^* mice was inhibited when sharing blood circulation with wild type animals, demonstrating the presence of an inhibitory molecule [62].

The observation of low levels of carboxylated anticalcifying factors in the blood of PXE and the report of PXE phenocopy due to a defect in vitamin K dependent γ-glutamyl carboxylase (GGCX) in humans [63], suggested a possible role for vitamin K in PXE. GGCX is required for the activation of matrix gla protein (MGP) by γ-glutamyl carboxylation with vitamin K2 as a co-factor [64]. The initial and intriguing hypothesis that ABCC6 could release vitamin K into the systemic circulation was rapidly discounted when it was shown that the supplementation of vitamin K2 failed to ameliorate the PXE phenotype in *Abcc6^−/−^* mice [65,66]. In 2014, Jansen et al. opened a new chapter in the pathophysiology of PXE [62]. In two pivotal studies [67,68], these authors demonstrated that expression of *ABCC6* in HEK cells was linked to a higher release of ATP in the culture media. Interestingly, a link between ABC transporters and extracellular levels of ATP is not new. Release of ATP has been also reported with cystic fibrosis trans membrane conductance regulator (CFTR, alias ABCC7) and *P*-glycoprotein (ABCB1) [69,70] suggesting at first that ABCC6 could extrude triphosphated compounds. In their cellular models, Jansen et al. failed to show a direct ABCC6-mediated ATP transport in vitro [68]. The fact that ABCC6 is involved in a pathway of extracellular nucleotide metabolism appears of paramount importance because it establishes a link between PXE and generalized arterial calcification of infancy (GACI) [71] or calcification of joints and arteries (ACDC) [72]. Indeed, the clinical manifestations linked to this pathway are substantial ectopic (cardiovascular) calcifications. The related diseases are caused by mutations in ectonucleotide pyrophosphatase/phosphodiesterase 1 (*ENPP1*) and in ecto-5’-nucleotidase (*NT5E*, alias *CD73*), which encode proteins involved in extracellular ATP and PPi metabolism (Table 2). This finding prompted the hypothesis that PXE patients could be deficient in PPi, which is a potent anti-calcifying molecule resulting from the hydrolysis of extracellular ATP [73]. Indeed, a 2.5 fold reduction of PPi levels in humans was observed in PXE patients and in *Abcc6*^−/−^ mice [67], whereas *Enpp1*^−/−^ mice have very low PPi levels [74]. Further, the cross-transplantation experiments between *Enpp1*^−/−^ and wild type mice showed that aortas of *Enpp1^−/−^* mice no longer showed calcification after transplantation into wild-type mice while allografts of wild-type mice calcified in *Enpp1^−/−^* mice [74]. It is now a matter of fact, that *Abcc6* deficiency in both local and distant cells/tissues is necessary to achieve the early onset and penetrant ectopic calcification observed in humans and mice [21]. Recently, Ziegler et al. showed that *Abcc6* conditional knockout in vascular endothelium, liver or the kidneys was not sufficient to promote calcification of the capsule of the vibrissae in mice [75]. In contrast, deletion of *Abcc6* in the liver led to mineralization after a period of one year. Most of the systemic PPi (60%) is believed to be produced by the liver in an ABCC6-dependent manner [33,67]. The fact that PPi levels only decrease after targeted disruption of *Abcc6* in the liver reinforces this hypothesis [75].

## 7. ABCC6 in the Context of Biomineralization

Inorganic pyrophosphate (PPi) is slightly absorbed by the intestinal epithelium [76]. Its normal fasting plasma level in humans is 2–4 μmol/L and it mainly depends on a balance between its cellular production and its hydrolysis [73]. PPi originates from the transport regulator protein encoded by *ANKH* [77] and from ATP degradation by ENPP1, which converts ATP into AMP and PPi. Physiological plasma levels of ATP are 0.02 to 0.2 μmol/L [78,79]. ATP, PPi and Pi are interdependent. PPi can be hydrolysed into Pi by Tissue Non-specific Alkaline Phosphatase (TNAP). Of note, adenosine resulting from the lysis of AMP by CD73 represses the expression of the gene encoding TNAP and is able to increase PPi levels [75]. Therefore, PPi levels depend on a balance between its production through ENPP1 and its degradation by the adenosine-regulated activity of TNAP. As a result, crossing *Abcc6^−/−^* mice with *Enpp1^−/−^* mice did not increase calcifications of the capsule of the vibrissae as observed in *Enpp1^−/−^* mice. In contrast, calcifications resulting from *Abcc6* inactivation were augmented by crossing *Abcc6^−/−^* mice with *Nt5e^−/−^* mice [75] and *Abcc6^−/−^* mice have reduced *Nt5e* expression in their arteries [15]. It should be remembered that other molecular mechanisms are able to mediate ATP release independently of ABCC6: vesicular exocytosis at nerve terminals, connexin hemichannel and pannexin channel activity [80]. These mechanisms are important because low PPi plasma levels of *Abcc6*^−/−^ mice and PXE patients [67] indicates that 40% of PPi comes from an ABCC6-independent ATP release. Despite overwhelming evidence linking PPi deficiency to the PXE phenotype, some concerns remain. As a matter of fact, the overexpression of the human *ENPP1* gene in *Abcc6^−/−^* mice led to incomplete reduction in mineralization despite increased PPi levels, suggesting that ABCC6 drives other cellular and/or molecular mechanisms resulting in the calcification phenotype in PXE [81].

## 8. A Role for ABCC6 into the Pathophysiology of Vascular Calcifications in Acquired Metabolic Diseases?

Calcifications of the arterial media (also termed mediacalcosis) are common features of CKD and osteoporosis. In contrast to PXE [16], mediacalcosis is linked to disturbed bone mineralization or bone turnover leading to increased risk of fracture and to the “bone-arterial calcification paradox” [17]. Remarkably, patients suffering from CKD have an increased rate of fracture [82,83] and patients affected by osteoporosis display more arterial calcifications [84] or develop them over time in parallel to loss of their mineral bone mass [85]. More than 10% of the general population suffers from CKD [86] and cardiovascular diseases represent the first cause of mortality in CKD [87,88] mostly associated to arterial calcifications. Arterial calcifications in CKD are not limited to mediacalcosis and atherosclerotic lesions are also present. A widely used marker of AC is the Agatston coronary artery calcification (CAC) score, measured with electron-beam computer tomography [89], which does not provide information on the spatial distribution of calcifications in the arterial wall (i.e. intimal versus medial layer). However, CAC score is a strong independent risk factor of cardiovascular mortality [90]. This score is remarkably high in CKD patients undergoing regular dialysis, even in children and in adults under the age of 30 [91,92]. The role of ABCC6 in CKD has not been addressed thoroughly yet. As a matter of fact, PPi/Pi ratio is low because Pi levels are high [93] and PPi levels are low [94,95] at least in advanced CKD stages. The observation of a lower hepatic expression of *Abcc6* in uremic rats [96] suggests that decreased PPi level observed in CKD could be caused by a decreased ATP-derived PPi production from the liver. TNAP react to uremic compounds because exposure of rat aortic ring to uremic plasma increases arterial TNAP activity and because *TNAP* expression increases in the arterial wall of the aorta of uremic rats following 5/6 nephrectomy [97]. This could lower local PPi level and favor AC [97]. Plasma TNAP activity is usually high in CKD following increased bone remodeling, which mainly results from secondary hyperparathyroidism. Remarkably, high TNAP activity is related to cardiovascular (CV) mortality in CKD after adjustment for other usual risk factors [98]. TNAP-driven increased PPi hydrolysis could augment AC and offer a rational explanation for this latter observation. Another frequent disease affecting 20% to 30% of the general population in Europe [99], which is associated with high level of AC is non-alcoholic fatty liver disease (NAFLD). Patients affected by NAFLD are at high risk for CAC independently from diabetes, gender and age [100,101]. NALFD patients primarily die from CV diseases [102,103,104]. Liver fibrosis seems of particular relevance regarding CAC and CV mortality. Indeed, CAC score is significantly higher in NAFLD patients with an intermediate to high fibrosis index than in those with a low fibrosis index [105]. Furthermore, liver fibrosis is independently associated with CAC score in NAFLD patients [106,107]. It has been reported that advanced fibrosis is also a significant predictor of mortality from CV disease in NAFLD patients [108]. To our knowledge, expression of *ABCC6* or the PPi/Pi ratio have not been examined in patients with liver fibrosis. However, it has been shown using liver ^32^P-NTP magnetic resonance imaging (MRI) that hepatic mitochondrial ATP homeostasis was impaired in patients with NAFLD [109,110]. Therefore, one might speculate that decreased ATP production from the liver could lower ABCC6-mediated extracellular ATP release and in turn reduce systemic PPi levels, which may provide an explanation for increased AC in NAFLD patients.

## 9. Therapeutic Perspectives

To date an etiologic treatment of the ABCC6 deficiency is still lacking. Therapeutics should aim at preventing hydroxyapatite deposits by increasing PPi plasma levels and/or at drawing calcium out of the calcifying lesions by lowering Pi levels. Phosphate binders are commonly used in CKD to lower Pi levels. They have been proposed for the treatment of PXE following a successful pilot study with aluminum hydroxide in humans [111]. However, a randomized controlled trial performed with sevelamer hydrochloride showed no improvement [112]. This unexpected result was attributed to the high magnesium (Mg) content in the placebo controls. Dietary supplementation with Mg in *Abcc6*^−/−^ mice was shown to contribute to reduce calcifications but the Mg concentrations used were more elevated than acceptable for human use [113]. Pharmacologic blockade of TNAP could be a valuable tool for limiting AC as this enzyme promotes the hydrolysis of PPi. However, the risk of bone demineralization, which is exemplified by the patients suffering from hypophosphatasia, may be a limitation [98]. PPi administration has been considered for the treatment of AC. Daily intraperitoneal injection almost completely inhibited calcification in *Abcc6*^−/−^ mice [114] and reduced the calcium content of the arterial wall of uremic rats without affecting bone mineralization [115]. Although originally thought to be completely hydrolyzed within the guts, oral gavage of mice with PPi was shown to significantly reduce calcification both in *Abcc6*^−/−^ and *Enpp1*^−/−^ mice [76]. Restoration of Abcc6 function with chemical chaperone 4-phenylbutyrate treatment in case of localization deficient disease caused by *Abcc6* mutants was also shown to be effective by rescuing injury-induced dystrophic cardiac calcifications [48,49]. Some bisphosphonate, non-hydrolysable analogs of PPi, prevent vascular calcifications induced by excess Vitamin D in rats [116] and reduce ectopic calcification in *Abcc6^−/−^* mice [117]. Soluble forms of ENPP1 where proposed as treatment to increase circulating PPi. Such treatment displayed efficacy to prevent calcification in *Enpp1*^−/−^ mice [118] and could theoretically be used in the context of ABCC6 deficiency. Finally, experimental data reported that vitamin K intake could improve soft tissue’s ectopic calcifications such as those induced by the use of anti-vitamin K drugs [119,120]. However, the addition of vitamin K2 to the diet of *Abcc6*^−/−^ mice did not significantly improved calcifications [65,66]. 

## 10. Conclusions

In conclusion, current results suggest that ABCC6-dependent plasmatic PPi levels are the major determinant of soft tissue mineralization in PXE, but also hint at a PPi independent unknown mechanism, by which ABCC6 prevents ectopic mineralization under physiologic conditions. ABCC6 involvement in the metabolism of the extracellular nucleotides, which play a central role for cellular energy, autocrine/paracrine and possibly remote signalizations, points to a possible role of purinergic signaling in the development of arterial calcifications in PXE patients. The understanding of the bio-mineralization process based on rare monogenic calcifying diseases sheds light on arterial calcifications and high cardiovascular mortality of CKD and NAFLD, which are far more frequent diseases, and could pave the way for therapeutic solutions.

## Figures and Tables

**Table 1:** Summary of PXE-associated *ABCC6* mutations.

Sequence Variations at Protein Level	Transport Activity	Wild-Type-Like Localization	Mutations Responding to 4-PBA	References
p.R1114P	Yes	No	Yes	[48,49]
p.S1121W	Yes	No	Yes	[48,49]
p.R1138Q	Yes	No	No	[48,50]
p.V1298F	No	Yes	Nd	[47,50]
p.T1301I	Yes	No	No	[48]
p.G1302R	No	Nd	Nd	[47]
p.R1314W	Yes	No	Yes	[47,48,49]
p.G1321S	No	No	Nd	[47,48,50]
p.R1339C	Nd	Not stable	No	[48,50]
p.Q1347H	Yes	No	Yes	[48,49]
p.R1459C	Yes	Yes	Nd	[48]

Summary of experimental evidences on the activity, localization and rescue of PXE-associated *ABCC6* mutations. In vitro and in vivo characterization of the given variants as well as rescue experiments of membrane trafficking deficient variants with the chemical chaperon molecule 4-phenylbutirate (4-PBA) is described in details within the listed articles. Nd: not determined. More information is available in Table S1.

**Table 2:** Overview of genetic calcifying diseases.

Human Disease	Protein	OMIM	Localization of Symptomatic Calcifications	Treatment
Pseudoxanthoma elasticum (PXE)	ATP-binding cassette transporter, subfamily C, member 6 (ABCC6)	264800	Skin, arteries	None
Craniometaphyseal dysplasia, autosomal dominant (CMDD) or Chondrocalcinosis 2 (CCAL2)	Inorganic pyrophosphate transport regulator (ANKH)	118600 or 123000	Cartilage (joints)	None
Generalized arterial calcification in infancy (GACI)	Ectonucleotide pyrophosphatase/phosphodiesterase 1 (ENPP1)	208000	Arteries	Bisphoshonate
Arterial calcification due to deficiency of CD73 (ACDC)	Ecto 5’ nucleotidase (NT5E) alias CD73	21288095	Arteries and distal joints	None
Keutel syndrome	Matrix Gla Protein (MGP)	245150	Cartilage (trachea, bronchiae, rib)	None
Juvenil Paget Disease/Hyperostosis corticalis deformans juvenilis	Osteoprotegerin (OPG)	239000	Bone	Bisphoshonate
Tumoral calcinosis, hyperphosphatemic	Klotho or fibroblast growth factor 23 (FGF23) or polypeptide N-acetylgalactosaminyl-transferase 3 (GALNT3)	211900	Arteries	None
Hutchinson–Gilford progeria syndrome (HGPS)	Laminin A (LMNA)	176670	Arteries, aortic valves	None
Fibrodysplasia ossificans progressiva (FOP)	Activin A receptor type 1 (ACVR1)	135100	Skeletal muscle, fascia, tendons and ligaments	Glucocorticoids, non-steroidal anti-inflammatory drugs
Coeliac disease with epilepsy and cerebral calcifications (CEC)	Unknown	226810	Brain (occipital area)	None
Idiopathic basal ganglia calcification (IBGC)	Sodium-dependent Pi co-transporter 2 (PiT-2) or platelet derived growth factor (PDGF) or (platelet derived growth factor receptor B) PDGFRB	158378 or 190040 or 173410	Brain (basal ganglia, thalamus, cerebellum)	None

## Supplementary Materials

Supplementary materials can be found at www.mdpi.com/1422-0067/18/9/1941/s1.
